# Penile Mondor's Disease Resulting From Forceful Condom Removal During Sexual Intercourse: A Case Report and Literature Review

**DOI:** 10.7759/cureus.50872

**Published:** 2023-12-20

**Authors:** Meshari A Alzahrani

**Affiliations:** 1 Urology, College of Medine, Majmaah University, Al Majmaah, SAU

**Keywords:** sexual intercourse, condom, mondor’s disease, penis, thrombophlebitis

## Abstract

Penile Mondor's disease, or dorsal vein thrombophlebitis, is vital for urologists to recognize. It causes pain and hardening in the penis due to triggers like trauma or neoplasms. Distinguishing it from similar conditions such as sclerosing lymphangitis and Peyronie’s disease is crucial. Penile Doppler ultrasound is the preferred diagnostic method. Providing reassurance can ease patient anxiety. This case report highlights a unique occurrence following forceful condom removal during sex, detailing symptoms, diagnosis, and successful treatment.

## Introduction

Mondor's disease (MD) manifests as palpable cord-like firm areas beneath the skin. Typically, MD is a benign condition that spontaneously resolves within four to eight weeks [1, 2]. The earliest instances of cord-like lesions on the chest wall were documented in the early 1850s, and in 1939, French surgeon Henri Mondor published a comprehensive case series detailing these lesions [1]. Since then, similar cord-like indurations have been noted in various body regions, such as the abdominal wall, groin, axilla, and penis. While there is no official classification, MD occurring on the anterolateral thoracoabdominal wall is commonly referred to as classic MD, while variations affecting other sites, such as the penis and axilla, are considered MD variants [3]. The specific variant affecting the penis is named penile Mondor's disease (PMD), initially reported by Helm et al in 1958 [4]. Another variant involving the axilla is termed axillary web syndrome (AWS), first described by Moskovitz et al in 2001 as a complication of axillary surgery [5]. While most MD cases are categorized as thrombophlebitis of the superficial vein, certain cases might involve lymphangitis or a combination of both.

The pathophysiology of MD remains unclear due to its rarity, despite the many years since its initial report. Moreover, MD affects diverse body regions, prompting patients to seek treatment from specialized clinics [6]. Previous reports suggest that the incidence of PMD is approximately 1.39%. This condition poses a significant risk to men aged between 20 and 40, especially those who do not experience pain [7-8]. PMD can be categorized into three clinical stages: acute, subacute-chronic, and repermeabilized "recanalization" [2,9-10]. The acute stage typically occurs in males aged 20 to 40 and manifests within 24 hours of prolonged sexual activity, likely due to vascular endothelial trauma [11]. Several studies indicate that the risk factors associated with PMD align with Virchow's triad which is defined by the following features: stasis, hypercoagulability, and vessel wall damage [7-8,12-13]. These factors encompass blood vessel wall damage caused by vigorous sexual activity, the use of vacuum erection devices, or penile trauma. Other factors, such as blood stasis resulting from prolonged erections, extended periods of sitting, and bladder overdistension, are also implicated. Furthermore, hypercoagulation, triggered by urogenital infections, prostate biopsies, and hematological disorders, constitutes another contributing factor to PMD [7]. The exact pathophysiology of this condition remains unknown; nevertheless, it is conjectured to be linked to mechanical stress, such as tugging and torsion of the dorsal vein of the penis following microtrauma or intense sexual activity [14]. Local infections arising from sexually transmitted diseases (STDs), venous obstruction due to bladder outlet obstruction, pelvic tumors (such as bladder or prostate cancer), metastatic pancreatic adenocarcinoma, and the use of vacuum erection devices all represent potential contributing factors [11,15]. Sexual trauma to the penile dorsal region, for instance, can lead to thrombophlebitis, and inflammatory processes can disrupt the penile venous network [16]. This case report details a novel instance of superficial thrombophlebitis of the dorsal vein of the penis resulting from the forceful removal of a condom during sexual intercourse.

## Case presentation

**Figure 1:**
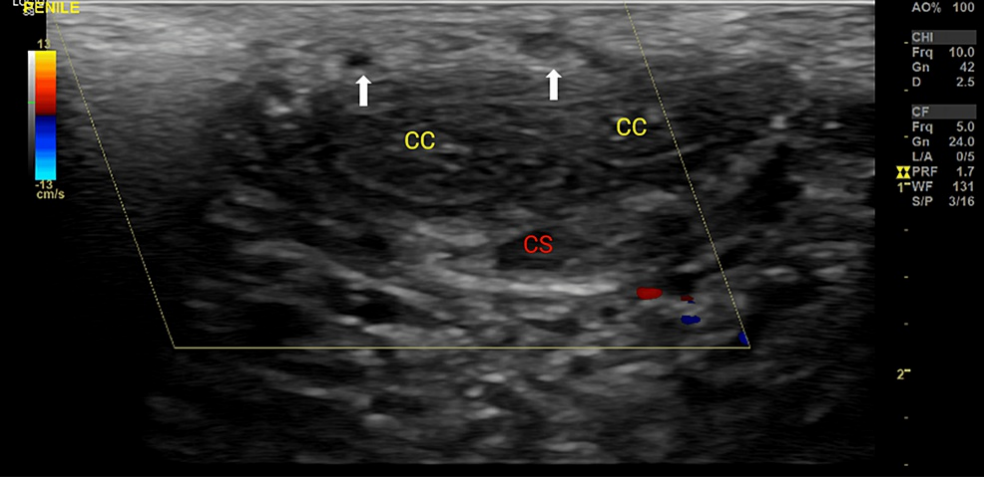
Penile color Doppler ultrasound: transverse section Yellow arrow: Corpus cavernosum (CC) of the penis; Red arrow: Corpus spongiosum (CS) of the penis. White arrow: Thrombosis of the superficial dorsal vein of the penis without flow.

**Figure 2:**
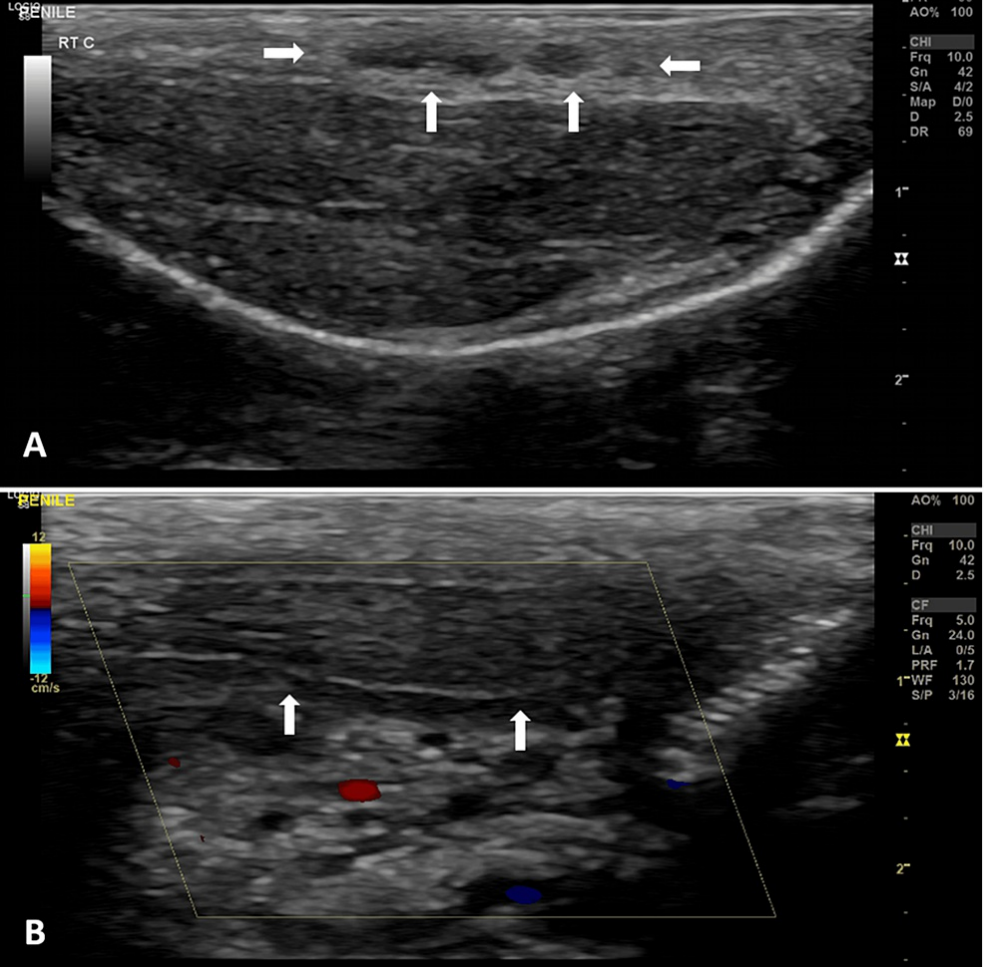
Penile ultrasound: longitudinal section Echogenic thrombus within the superficial dorsal vein of the penis (white arrows in A, B) with no flow on the Penile Color Doppler Ultrasound (white arrow in B).

A 41-year-old male, a smoker with no prior medical history, presented at the andrology clinic with a 3-day history of penile pain. He reported experiencing severe penile pain immediately after forcefully and aggressively removing a condom with rapid withdrawal, accompanied by strong pulling of his penis during sexual intercourse. This was followed by a swift detumescence. He noted that there were no incidents of penile buckling, popping, or swelling. The patient denied experiencing fever, chills, penile or urethral discharge, or any lower urinary tract symptoms. He also denied any recent history of STDs, urinary tract infection (UTI), or the use of phosphodiesterase 5 inhibitors (PDE5i). During the genital examination, there were no indications of erythema or swelling. A palpable, thin, cord-like structure was observed within the superficial dorsal vein of the penis, running along the dorsal aspect of the penis. This structure exhibited no tenderness. A transverse (Figure 1) and longitudinal (Figure 2) grayscale ultrasound of the dorsal vein of the penis was performed. Penile ultrasound was conducted while the penis was flaccid. Both the corpora cavernosa and corpus spongiosum appeared intact without any evident defects. There were no significant swellings or apparent penile ruptures. A small internal echogenic focus was detected within the superficial dorsal vein, with absent flow (Figure 1 and Figure 2A, 2B), which suggested a diagnosis of PMD. The patient was informed of this finding. Reassurance and conservative management were recommended, including the application of warm compresses, nonsteroidal anti-inflammatory medications, and sexual abstinence for two weeks to enhance recovery. Follow-up revealed complete symptom resolution within 8 weeks, and the patient resumed sexual activity after 2 weeks of abstinence. Till date, to the moment of writing the manuscript, at 6 months follow-up, the patient was asymptomatic, with complete resolution of symptoms that presented earlier, with normal sexual activity.

## Discussion

There have been several reports suggesting that sexual activity can induce PMD. One study documents the case of a young male who developed PMD after he engaged in masturbation three times a day over the course of seven days [17]. PMD can also arise from vigorous sexual intercourse, as reported in various case studies [18, 19]. Another report described the subsequent formation of a preputial leaf abscess two weeks after the onset of PMD and subcutaneous lymphangitis resulting from extensive sexual activity [2]. Although its pathophysiology remains incompletely understood, several known etiological factors exist [20]. Numerous potential causes have been identified in various studies, as summarized in Table 1 [3, 7, 21-35]. Our case report is the first in the literature to document PMD resulting from forceful condom removal during sexual intercourse. In this scenario, forcefully removing the condom might be linked to aggressive sexual behavior and could be considered a potential risk factor for PMD. In PMD, induration arises on the dorsal and dorsolateral parts of the penis due to the involvement of the inferior component of the abdominal wall's superficial vein system [12]. The circumflex vein can also be affected, which some authors regard as an uncommon manifestation of PMD [36].

**Table 1:** Summary of reported etiology for Penile Mondor's Disease.

Etiology category	Reported etiology
Infectious	Local infection:
	Candida infection.
	Sexually transmitted disease
	Syphilis.
	HIV.
	Distant infection:
	COVID-19 (SARS-CoV-2).
Traumatic	Penile trauma.
	Body budling exercises.
Sexual activity	Frequent or excessive sexual activity.
	Intense or vigorous sexual activity.
	Use penile constrictive element.
	Use a penile vacuum device.
	Forceful Condom removal.
Medications	Phosphodiesterase 5 Inhibitors.
	Intracavernous drugs.
	Abuse of certain intravenous drugs.
Surgical	Varicocelectomy.
	Orchiopexy.
	Inguinal hernia repair.
	Prostate biopsy
Healing disorders	Peyronie's disease.
Hematological disorder	Thrombophilia.
Vascular disorder	Behçet’s disease.
Malignancy	Pelvic cancer.
	Metastatic pancreas cancer (adenocarcinoma).
	Migratory phlebitides due to paraneoplastic syndromes.
Others	Venous occlusion caused by bladder distension.

A medical history and physical examination are employed to diagnose PMD. Patients typically exhibit a rope-like cord on the dorsal side of the penis. This cord represents a thrombosed dorsal vein that has become swollen and adhered to the skin. Often, the lesion will extend superiorly into the suprapubic area. The vein may appear enlarged and red. Patients commonly report significant discomfort, which can manifest intermittently or persistently. In some cases, affected individuals might also experience irritative urinary symptoms [37, 38]. In our presented case, the patient's chief complaint was the sensation of a rope-like cord on the dorsal side of the penis three days after a forceful condom removal incident. Previous literature reported condom catheter-related injuries and penile skin erosion for male patients with urinary incontinence, bedridden patients, and the geriatric population [39, 40]. Trauma caused by condoms during sexual activity is seldom documented. To the best of our knowledge, our study represents the first instance in English literature where forceful condom removal has been linked to PMD.

Testing for STDs should be conducted in patients with PMD, as several reports have indicated an association between PMD and syphilis and HIV-positive patients [30, 31]. The largest known series of patients visiting an STD clinic reported a PMD incidence of 1.39% [26]. Distinct from PMD, Peyronie's disease is characterized by subcutaneous sclerosing lesions with a distinctive curved shape and a history of penile trauma [32]. Additionally, two case reports have suggested a link between SARS-CoV-2 infection and PMD due to COVID-19's documented impact on thrombosis pathophysiology [33-35]. Moreover, in men with erectile dysfunction and a low International Index of Erectile Function (IIEF-5) score, PMD may exacerbate symptoms [41].

Evaluation of the body's surface necessitates the use of a linear-type transducer. Grayscale ultrasonography is effective in detecting thrombosed superficial veins, which appear as uncompressible subcutaneous tube structures with anechoic or hypoechoic contents [42]. When flow signals are absent within the tubular structure, color Doppler sonograms can be employed. Color Doppler is also useful for monitoring lesions, as flow recovery implies vein recanalization [43]. For a more comprehensive view of the venous system, magnetic resonance angiography (MRA) is beneficial. It may be particularly helpful in cases such as PMD following a prostate biopsy, where it can rule out hematoma or iatrogenic lesions [44]. However, in our case, ultrasound was sufficient as the diagnostic tool.

A diagnostic and management approach for PMD has been proposed [7]. This involves initially assessing underlying concerns, cancer probability, and the potential for Peyronie's disease. Subsequent monitoring of the probable PMD patient for four to eight weeks is recommended. Prophylactic antibiotics are not necessary in such cases. Conservative treatments include sexual abstinence, heparin ointment, and oral nonsteroidal anti-inflammatory medications (NSAIDs), although these are empirical. If no resolution occurs, options include starting anticoagulation and going for further investigation such as ultrasonography, and biopsy [8, 26]. A study reported treatment with oral antiphlogistic therapy (indomethacin) combined with a topical dressing containing heparin ointment (10,000 IU) produced favorable results for cases presented with PMD showing a success rate for conservative therapy of 92% [8]. Surgical management, involving excision of the entire thrombosed vein and advising sexual abstinence for 6 weeks, has been reported. This approach led to symptom improvement [45]. In our case, our management protocol included advising sexual abstinence for 2 weeks and initiating NSAIDs (Etoricoxib 90 mg tablet every 24 hours for 2 weeks), along with warm compression as needed. Follow-up revealed complete symptom resolution within 8 weeks, and the patient resumed sexual activity after 2 weeks of abstinence. In numerous cases, recovery occurs within 4 to 6 weeks, with vessel permeability restored in 9 weeks, even without medical intervention [23]. The long-term prognosis of MD remains underreported. One study found excellent clinical outcomes without recurrence for nearly three years in four patients with chest MD, while another study noted recurrence in 3 out of 23 chest MD patients over nine years [46]. However, only a small percentage of PMD patients experience persistent pain and priapism, and most are symptom-free [47]. Regarding functional prognosis, a study involving 30 PMD patients found no lasting penile deformity or erectile dysfunction after 2 months of follow-up [48]. In our case, upon a 6-month follow-up, the patient experienced the complete alleviation of symptoms associated with PMD, showing no noticeable penile deformity, and successfully resumed sexual activity. The connection between MD and superficial thrombophlebitis at other sites is unclear, but these lesions are considered early indicators of widespread thrombophlebitis [49]. Therefore, the prognosis of secondary MD depends on the prognosis of the underlying disease [3].

## Conclusions

PMD is an infrequent condition characterized by the development of cord-like indurations beneath the skin of the penis. It pertains to a pathology of the superficial dorsal penile vein and should be considered within the scope of diagnosing penile ailments. Thrombophlebitis is a common occurrence in both MD and PMD patients. Generally, MD is a self-limiting condition that tends to resolve within a period of four to eight weeks without necessitating medical intervention. While some MD cases arise without any underlying cause, others might be linked to conditions such as vasculitis, hypercoagulation, or even cancer. Physicians must adeptly identify MDs, evaluate the potential presence of an underlying condition, and avoid unnecessary invasive tests or treatments.

## References

[1] Hokama A, Fujita J (2010). Mondor disease: an unusual cause of chest pain. South Med J.

[2] Ozkara H, Akkuş E, Alici B, Akpinar H, Hattat H (1996). Superficial dorsal penile vein thrombosis (penile Mondor's disease). Int Urol Nephrol.

[3] Amano M, Shimizu T (2018). Mondor's disease: A review of the literature. Intern Med.

[4] Helm JD Jr, Hodge IG (1958). Thrombophlebitis of a dorsal vein of the penis: report of a case treated by phenylbutazone (butazolidin). J Urol.

[5] Moskovitz AH, Anderson BO, Yeung RS, Byrd DR, Lawton TJ, Moe RE (2001). Axillary web syndrome after axillary dissection. Am J Surg.

[6] Farrow JH (1955). Thrombophlebitis of the superficial veins of the breast and anterior chest wall (Mondor's disease). Surg Gynecol Obstet.

[7] Manimala NJ, Parker J (2015). Evaluation and treatment of penile thrombophlebitis (Mondor's disease). Curr Urol Rep.

[8] Al-Mwalad M, Loertzer H, Wicht A, Fornara P (2006). Subcutaneous penile vein thrombosis (penile Mondor's disease): Pathogenesis, diagnosis, and therapy. Urology.

[9] Swierzewski SJ 3rd, Denil J, Ohl DA (1993). The management of penile Mondor's phlebitis: Superficial dorsal penile vein thrombosis. J Urol.

[10] Tanii T, Hamada T, Asai Y, Yorifuji T (1984). Mondor's phlebitis of the penis: A study with factor VIII related antigen. Acta Derm Venereol.

[11] Nazir SS, Khan M (2010). Thrombosis of the dorsal vein of the penis (Mondor's disease): A case report and review of the literature. Indian J Urol.

[12] Mayor M, Burón I, de Mora JC, Lázaro TE, Hernández-Cano N, Rubio FA, Casado M (2000). Mondor's disease. Int J Dermatol.

[13] Kervancioglu S, Ozkur A, Bayram MM (2005). Color Doppler sonographic findings in penile fracture. J Clin Ultrasound.

[14] Ouattara A, Paré AK, Kaboré AF (2019). Subcutaneous dorsal penile vein thrombosis or penile Mondor's disease: A case report and literature review. Case Rep Urol.

[15] Polat H, Cift A, Gulacti U, Benlioglu C (2015). The damage of penile Doppler ultrasonography in diagnosis of penile Mondor's disease: A report of two cases. Urol Case Rep.

[16] Rodríguez FO, Parra ML, Gómez SCC, Martín B, Escaf BS (2006). Thrombosis of the dorsal penis vein (of Mondor's phlebitis). Presentation of a new case. [Article in Spanish]. Actas Urol Esp.

[17] Sheikh MM, Jeelani HM, Farooqi A, Riaz A (2020). Penile Mondor's disease: Rare association with excessive masturbation. Cureus.

[18] Foresti M, Parmiggiani A (2020). Penile Mondor's disease: Imaging in two cases. J Radiol Case Rep.

[19] Aydin M, Tuncay TAŞ, Gursoy G (2012). Penile Mondor's disease induced by vigorous sexual activity: Two cases, two different approaches. Turkiye Klinikleri J Med Sci.

[20] Özkan B, Coşkuner ER (2022). What we know about penile Mondor's disease. Sex Med Rev.

[21] Horn AS, Pecora A, Chiesa JC, Alloy A (1985). Penile thrombophlebitis as a presenting manifestation of pancreatic carcinoma. Am J Gastroenterol.

[22] Bennett RG, Leyden JJ, Decherd JW (1973). The heroin ulcer. New addition to the differential diagnosis of ulcers of the penis. Arch Dermatol.

[23] Öztürk H (2014). Penile Mondor's disease. Basic Clin Androl.

[24] Walsh JC, Poimboeuf S, Garvin DS (2014). A common presentation to an uncommon disease. Penile Mondor's disease: A case report and literature review. Int Med Case Rep J.

[25] Kumar B, Narang T, Radotra BD, Gupta S (2005). Mondor's disease of penis: A forgotten disease. Sex Transm Infect.

[26] Wild J, Wilson L, Bajaj M (2020). Penile Mondor's disease- An understated entity. Urol Case Rep.

[27] Griger DT, Angelo TE, Grisier DB (2001). Penile Mondor's disease in a 22-year-old man. J Am Osteopath Assoc.

[28] Kartsaklis P, Konstantinidis C, Thomas C, Tsimara M, Andreadakis S, Gekas A (2008). Penile Mondor's disease: A case report. Cases J.

[29] Zor M, Tahmaz L, Basal S, Irkilata HC (2009). Penile Mondor's disease in a 32-year-old man: Case report. Turkiye Klinikleri J Med Sci.

[30] Chakra MA, Roux S, Peyromaure M, Delongchamps NB, Bailly H, Duquesne I (2022). An unusual presentation of penile Mondor's disease in an HIV-positive patient. Ann R Coll Surg Engl.

[31] Rani R (2009). Mondor's disease of the penis associated with primary syphilis. Int J STD AIDS.

[32] Hakim LS (2002). Peyronie's disease: An update. The role of diagnostics. Int J Impot Res.

[33] Bagheri SM, Tabrizi Z (2021). Deep dorsal penile vein thrombosis in a patient with COVID-19 infection: A rare complication and the first reported case. Clin Case Rep.

[34] Balawender K, Pliszka A, Surowiec A, Rajda S (2022). COVID-19 infection as a new risk factor for penile Mondor disease. BMC Urol.

[35] Eren MT, Özveri H, Kurtoğlu H (2021). Penile Mondor's in a COVID-19 patient on prophylactic anti-thrombosis with rivaroxaban: A case report. Afr J Urol.

[36] Arora R, Sonthalia S, Gera T, Sarkar R (2015). Atypical penile Mondor's disease - Involvement of the circumflex vein. Int J STD AIDS.

[37] Sasso F, Gulino G, Basar M, Carbone A, Torricelli P, Alcini E (1996). Penile Mondors' disease: An underestimated pathology. Br J Urol.

[38] Thomazeau H, Alno L, Lobel B (1983). Thrombosis of the dorsal vein of the penis. Apropos of 2 cases. [Article in French]. J Urol (Paris).

[39] Zaghbib S, Chakroun M, Saadi A (2019). Severe penile injury due to condom catheter fixed by a rubber band: A case report. Int J Surg Case Rep.

[40] Sinha AK, Kumar N, Kumar A, Singh S (2018). Condom catheter induced penile skin erosion. J Surg Case Rep.

[41] Durmus E, Ok F (2022). Could penile Mondor's disease worsen symptoms in patients with erectile dysfunction?. J Invest Surg.

[42] Yanik B, Conkbayir I, Oner O, Hekimoğlu B (2003). Imaging findings in Mondor's disease. J Clin Ultrasound.

[43] Avery LL, Scheinfeld MH (2013). Imaging of penile and scrotal emergencies. Radiographics.

[44] Boscolo-Berto R, Iafrate M, Casarrubea G, Ficarra V (2009). Magnetic resonance angiography findings of penile Mondor's disease. J Magn Reson Imaging.

[45] Gahlawat S, Gupta D, Kumar A, Sood R (2019). Surgical management of penile Mondor’s disease: Case report and brief review of literature. Indian J Case Rep.

[46] Salemis NS, Merkouris S, Kimpouri K (2011). Mondor's disease of the breast. A retrospective review. Breast Dis.

[47] Zidani H, Foughali M, Laroche JP (2010). Superficial venous thrombosis of the penis: penile Mondor's disease? A case report and literature review [Article in French]. J Mal Vasc.

[48] Özkan B, Coskuner ER, Turk A, Akkus E, Yalçın V (2015). Penile Mondor disease and its effect on erectile function: Results of 30 patients. Urology.

[49] Luis Rodríguez-Peralto J, Carrillo R, Rosales B, Rodríguez-Gil Y (2007). Superficial thrombophlebitis. Semin Cutan Med Surg.

